# Histologic and Genetic Advances in Refining the Diagnosis of “Undifferentiated Pleomorphic Sarcoma”

**DOI:** 10.3390/cancers5010218

**Published:** 2013-02-22

**Authors:** Fergal C. Kelleher, Antonella Viterbo

**Affiliations:** 1 Sarcoma Service, Department of Medical Oncology, Peter Mac Callum Cancer Centre, Melbourne, Victoria, VIC8006, Australia; 2 Department of Medical Oncology, St. Vincent’s University Hospital, Dublin 4, Ireland; 3 St. Andrea University Hospital, Rome 000189, Italy; E-Mail: antonella.viterbo@libero.it

**Keywords:** undifferentiated pleomorphic sarcoma, malignant fibrous histiocytoma, gene expression arrays, lineage differentiation

## Abstract

Undifferentiated pleomorphic sarcoma (UPS) is an inclusive term used for sarcomas that defy formal sub-classification. The frequency with which this diagnosis is assigned has decreased in the last twenty years. This is because when implemented, careful histologic assessment, immunohistochemistry, and ultra-structural evaluation can often determine lineage of differentiation. Further attrition in the diagnostic frequency of UPS may arise by using array-comparative genomic hybridization. Gene expression arrays are also of potential use as they permit hierarchical gene clustering. Appraisal of the literature is difficult due to a historical perspective in which specific molecular diagnostic methods were previously unavailable. The American Joint Committee on Cancer (AJCC) classification has changed with different inclusion criteria. Taxonomy challenges also exist with the older term “malignant fibrous histiocytoma” being replaced by “UPS”. In 2010 an analysis of multiple sarcoma expression databases using a 170-gene predictor, re-classified most MFH and “not-otherwise-specified” (NOS) tumors as liposarcomas, leiomyosarcomas or fibrosarcomas. Interestingly, some of the classifier genes are potential molecular therapeutic targets including Insulin-like growth factor 1 (IGF-1), Peroxisome proliferator-activated receptor γ (PPARγ), Nerve growth factor β (NGF β) and Fibroblast growth factor receptor (FGFR).

## 1. Introduction

The undifferentiated pleomorphic sarcoma (UPS, previously MFH) tumour category is a heterogeneous group of disorders. It is mainly composed of mesenchymal lineages that belong to established sarcoma subgroups such as liposarcoma, fibrosarcoma and leiomyosarcoma. This publication deliberately preserves the terminology of the original cited literature to emphasise the historic trends. Improvements in histologic practices and institution of gene expression clustering methods can frequently establish the true diagnostic assignment. Discrimination between publications that use genetic methods to sub-classify sarcoma series rather than publications exclusively restricted to cases of UPS is also required.

## 2. Sarcoma

Sarcomas arise from mesenchymal stem/progenitor cells and account for ~1% of all malignancies. There are two main molecular sub-classes of sarcoma. The first is characterized by a single recurrent genetic aberration, such as somatic mutations of the v-kit Hardy-Zuckerman 4 feline sarcoma viral oncogene homolog (KIT) proto-oncogene in gastrointestinal stromal tumors or chromosomal translocations causing fusion genes. These would include the translocated in liposarcoma; C/EBP-homologous protein (TLS-CHOP) fusion transcript in myxoid/round cell liposarcoma or synovial sarcoma translocation; synovial sarcoma X (SYT-SSX1) or SYT-SSX2 [1,2]. Many of the sarcomas characterised by tumor-specific chromosomal translocations tend to occur in younger patients. The second subtype includes de-differentiated or pleomorphic liposarcoma, leiomyosarcoma, fibrosarcoma and MFH. The sarcomas in this subdivision have numerous non-recurrent chromosomal aberrations leading to complex karyotypes [3]. The genomic instability leading to these complex karyotypes possibly arises because of loss of telomeres. Approximately 85% of cancers lengthen telomeres by activating the enzyme telomerase, whereas 15% use alternative telomere lengthening, a recombination method [4]. Telomeres occupy the end region of chromosomes and have extended repeats of the nucleotide sequence TTAGGG. Telomeres shorten with successive cell divisions unless replaced by the enzyme telomerase. In cancer the loss of the protective telomere chromosomal cap leads to intra- and inter-chromosomal bridges at mitosis. All osteosarcomas have complex karyotypes and 50% depend on alternative telomere lengthening to maintain telomeres [5,6].

In 2010 a different sub-grouping system for sarcomas was devised that consists of four categories, assigned based on clinical, pathologic and molecular features [7]. In that system, *category one sarcomas*, consist of non-pleomorphic tumors with pathogenomic molecular events, e.g., gastrointestinal stromal tumors, *KIT* or platelet-derived growth factor receptor, alpha polypeptide (*PDGFRA*) mutations; dermatofibrosarcoma protuberans, t(17;22)(q22;q13), ring chromosomal alterations, involved genes *COL1A1-PDGFRB*; and Ewing’s sarcoma, chromosomal translocation t(11:22; q24; q12), in 85% of cases, involved genes *FLI1-EWS*. *Category two sarcomas*, usually affect younger patients and are non-pleomorphic tumors for which pathogenomic molecular events have not been found to date, e.g., adamantinoma or chordoma. *Category three* sarcomas, are pleomorphic with some defined molecular changes and complex karyotypes including, dedifferentiated liposarcoma, malignant peripheral nerve sheath tumor (MPNST) and myxoinflammatory fibroblastic sarcoma. They have consistent molecular events, e.g., dedifferentiated liposarcoma, cyclin dependent kinase 4/mouse double minute 2 homolog (CDK4/MDM2) amplification; MPNST, deletion of NF1; myxo-inflammatory fibroblastic sarcoma, t(1;10) and 3p amplification. *Category 4* consists of pleomorphic sarcoma with complex karytotypes and gene expression profiles, e.g., MFH, leiomyosarcoma and osteosarcoma.

## 3. MFH, Morphologic Reassessment, Immunohistochemistry and Ultra-Structural Re-Evaluation

MFH was previously considered the most common soft tissue sarcoma in adults. In 1992 however, Fletcher published a retrospective series of 159 cases diagnosed as pleomorphic sarcoma from the files of the Histopathology Department, St. Thomas’s Hospital, London [8]. These tumors were re-assessed morphologically, using meticulous tumor sampling, immunohistochemistry, and ultra-structurally. Of these cases, 63% were discovered to be specific sarcoma subtypes other than MFH, 12.5% were non-mesenchymal neoplasms, including carcinomas, lymphomas, and melanoma. Another 26% were unclassifiable. Thirteen percent of the total and 50% of this unclassifiable group were small biopsies or sub totally necrotic. Only 13% of the cases in this retrospective series were truly eligible to be diagnosed as MFH. Interestingly, five of six cases of extraskeletal osteosarcoma in this series were indistinguishable from MFH, except for the presence of malignant osteoid in small areas. A definable line of differentiation that permitted exclusion of the diagnosis of MFH, was found in 74% of cases.

It was inferred that MFH comprises a heterogeneous group of poorly differentiated neoplasms. At that time two possible origins of UPS were considered likely: (i) that it arises from a primitive pluripotential mesenchymal cell that demonstrates different extents of differentiation; (ii) that it is a nonspecific entity of poorly differentiated neoplasms of different types. A temporal attrition in the diagnostic frequency of MFH was anticipated and did occur because of the discovery of new tumour antigens, recognition of new cytogenetic abnormalities and gene expression profile technologies.

In a separate follow-up study in 2001, Fletcher led a group which again re-classified 100 tumors of the trunk wall and extremity that had been primarily classified as MFH [9]. The thigh was the most common tumor location at 31%. The rate of overall metastasis-free survival was 0.64. A specific line of differentiation was either proven or strongly suggested in 84% of cases; myxofibrosarcoma was the most common (22% of total), followed by leiomyosarcoma at 20%. The study findings are documented in Table 1.

Divergent clinical outcomes occur within pleomorphic sarcomas. As an exemplary example, when this study was published in 2001, dedifferentiated liposarcomas had a metastasis risk of less than 25%, whereas the 5-year rate of metastasis for high-grade myxofibrosarcoma was 35–40% [10,11,12,13].

**Table 1:** Diagnosis after re-evaluation of 100 soft tissue sarcomas of the extremities and trunk wall. Cases initially diagnosed as MFH in 1964–1997. Data derived from the data registry of the Musculoskeletal Tumour Center, Lund, Sweden.

Tumor		Number of patients
Myxofibrosarcoma		29
	High grade	23
	Intermediate grade	3
	Low grade	3
Myogenic sarcoma		30
	High grade leiomyosarcoma	20
	Pleomorphic rhabdomyosarcoma	1
	High grade myogenic sarcoma	9
NOS		
Liposarcoma		4
	Pleomorphic liposarcoma	3
	Low grade liposarcoma	1
Malignant peripheral nerve sheath tumor		2
	Low grade	1
	High grade	1
Soft tissue osteosarcoma		3
Malignant mesenchymoma		2
Myofibroblastic sarcoma		11
High grade sarcoma without specific line of differentiation		2
Possibly high grade myofibroblastic sarcoma		9
Dermatofibrosarcoma protuberans with fibrosarcomatous change		1
Low grade fibromyxoid sarcoma		1
High-grade sarcoma with specific line of differ.		16
	Pleomorphic	12
	Spindle-cell	2
	Spindle cell/myxoid	1
	Pleomorphic/myxoid	1
	Sarcomatoid carcinoma	1
Total of all tumors		100

## 4. Sarcoma Series and Gene Expression Profiles

MFH does not exhibit histologic evidence of differentiation and possibly is a collection of different poorly differentiated sarcoma types. There are however four descriptive histologic MFH subtypes: myxoid, storiform & pleomorphic, giant cell and inflammatory. Additionally an analysis in 2002 of the gene-expression patterns of 41 soft tissue sarcomas failed to find a clear distinction between the genes of MFH, liopsarcoma and leiomyosarcoma [14].

In 2007 an independent study was published of gene expression analysis of 105 samples representing 10 types of soft tissue tumors [15]. The spindle and pleomorphic sarcomas (e.g., myxofibrosarcoma, leiomyosarcoma, dedifferentiated liposarcoma, fibrosarcoma, malignant peripheral nerve sheath tumor and MFH) were found to have a similar gene expression profiles compared to other tumors (e.g., myxoid/round cell liposarcoma, synovial sarcoma, lipoma and well differentiated liposarcoma). Sixty four cases of the spindle cell and pleomorphic sarcoma were evaluated and residual gene heterogeneity persisted with respect to MFH. In retrospect three of the 21 MFH samples were misclassified when high gene expression was applied to tumor type classification. However, all had marked histologic pleomorphism, implying that a diagnosis of MFH was appropriate at the time. Of the remaining 18 cases, 12 had moderate gene expression similarity to the remaining five sarcoma subtypes.

Therefore taxonomy of MFH requires gene expression profiling to eliminate contamination with other sarcoma subtypes due to histologic pleomorphism. The remaining MFH cases may have features suggestive of other sarcoma types (myxofibrosarcoma, leiomyosarcoma, dedifferentiated liposarcoma, fibrosarcoma, malignant peripheral nerve sheath tumor) but insufficient for definitive classification.

**Figure 1:**
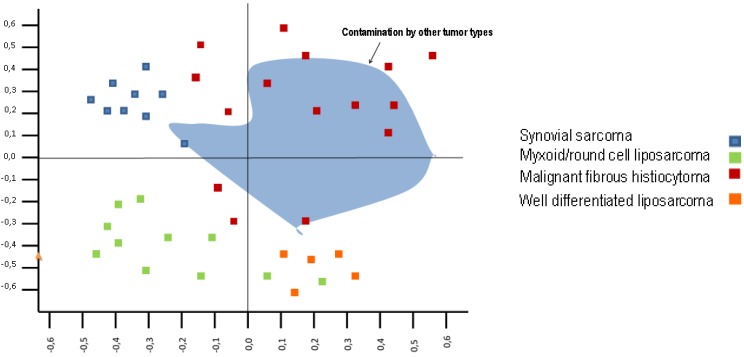
Principle component analysis of gene expression of a series of 10 types of soft tissue tumor (synovial sarcoma, myxoid/ round cell liposarcoma, well-differentiated liposarcoma, dedifferentiated liposarcoma, lipoma, myxofibrosarcoma, leiomyosarcoma, malignant peripheral nerve sheath tumor, fibrosarcoma, MFH). Gene expression overview of the 105 soft tissue tumors with 12,599 probe sets. Each square represents an individual analysed tumor; Nakayama *et al*. [15].

The results of the principle component analysis (which is a counterpart of Euclidean distance measurement for clustering analysis, with the first principle component (PC1) being the *x-axis* and the second principle component (PC2) being the *y-axis*) suggested that: (i) PC1 was associated with the difference between synovial sarcoma + myxoid/round cell liposarcoma and spindle cell pleomorphic sarcoma and, (ii) PC2 was associated with adipocytic differentiation (Figure 1). The uncertainty remains if MFH is a unifying morphologic classification of a variety of poorly differentiated well recognised sarcoma subtypes or whether the MFH designation also contains some sarcoma groups still to be described as specific entities.

In the Nakayama study a third of MFH had a gene expression profile that was similar to myxofibrosarcoma. The investigators hypothesised that a large proportion of MFH may actually be a pleomorphic subtype of myxofibrosarcoma. Clinically this tumor has a high local recurrence rate and is frequent in older adults [10]. The genes, WNT1 inducible signaling pathway protein 2 (WISP2), G protein-coupled receptor 64 (GPR64) and Tenascin XB (TNXB) were found to be upregulated in myxofibrosarcoma compared with other spindle cell and pleomorphic sarcomas. WISP2 belongs to the WNT1 (WNT name, is a portmanteau of Int and Wg; wingless) inducible signalling pathway, a constituent of the connective tissue growth factor family. WISP is a cell and matrix associated proteins with roles including differentiation and survival. GRP64 is a receptor on the epididymis of uncertain importance in cancer, and TNXB is a member of the tenascin family of extracellular matrix proteins [16]. Differential gene expression allowed for the reclassification of 70% of MFH into the five sarcoma types: myxofibrosarcoma, leiomyosarcoma, dedifferentiated liposarcoma, fibrosarcoma, and malignant peripheral nerve sheath tumor.

Gene expression microarray analysis was performed on 181 tumors which represented 16 classes of bone and soft tissue sarcoma in a 2005 study [17]. Leiomyosarcoma, liposarcoma and malignant fibrous histiocytoma subgroups were identified. Unsupervised hierarchical clustering was undertaken using the 1,527 most variably expressed genes in the data set. The dendrogram had two major braches. The first containing tight clusters of sarcoma types such as; osteosarcoma, rhabdomyosarcoma, Ewing’s sarcoma, synovial sarcoma, dermatofibrosarcoma protuberans, gastrointestinal stromal tumors and myxoid liposarcomas. The second branch contained subtypes that clustered more loosely; leiomyosarcoma, MFH and the remaining liposarcomas. The liposarcoma group contained two tumour types defined by gene expression data; those that clustered tightly and those that intermingled with cases of MFH. The majority of this later group were classified as higher grade. Three groups, peripheral nerve sheath tumors (malignant peripheral nerve sheath tumors and benign schwannomas), fibrosarcomas and miscellaneous tumors failed to form clusters distinct from the other groups.

## 5. Undifferentiated Pleomorphic Sarcoma and Gene Expression

Some MFH tumors are immunohistochemically positive for muscle associated proteins. In 2005, Baird *et al*. found that in an unsupervised clustering dendrogram, the branch adjacent to MFH contained leiomyosarcoma. Within the MFH class there were two almost equally sized groups. These groups were subjected to gene ontology and weighted gene analysis. A compendium of 279 differentially expressed genes (*p* < 0.001) was generated that had 41 up-regulated genes in the first group and the remaining 238 genes in the second group. The first group had genes associated with a muscle profile. The 10 most significantly expressed genes included *myosin X*, *tenascin C* and *sarcoglycan β*. The second group was dominated by immune regulatory genes, with the five most highly weighted genes being HEM1, MX1, DAP10, PLCG2, and FOLR3. Using an ontologic gene classifier the genes in the first group mapped to the ontologic family of “muscle activity” whereas those of the second group mapped to the ontologic family of “immune activity and cell adhesion”. Myogenic differentiation in MFH and undifferentiated sarcomas is correlated with a poor prognosis [9].

The adverse prognosis of myogenic differentiation is vicariously supported by finding from another study [18]. Gene set analysis and hierarchical clustering established differences in the time to development of metastatic disease for high grade soft tissue sarcomas. Seventy three cases of high grade pleomorphic soft tissue sarcomas, primary undifferentiated soft tissue sarcoma and leiomyosarcoma were assessed. A comparison of time to metastasis, between leiomyosarcoma and undifferentiated pleomorphic sarcoma sample sets established a 0.65 probability of no metastasis at 5 years for undifferentiated pleomorphic sarcoma and 0.43 for leiomyosarcoma (*p* = 0.02).

## 6. Undifferentiated Pleomorphic Sarcoma and Tumor Location

In certain circumstances the anatomic location in which an UPS arises can be informative as to it’s true nature; one may refer to Table 2 [19,20,21,22,23,24,25].

**Table 2:** Histologic subtypes and individualized frequencies of retroperitoneal sarcomas diagnosed in 2003.

*Histologic subtype*	*Frequencies*
MFH	7–30%
Poorly differentiated sarcomas (MFH, fibrosarcoma, malignant hemangiopericytoma, unclassified sarcoma)	16–50%
Liposarcoma	20–40%
Leiomyosarcoma	10–30%

Extensive tissue sampling, immunohistochemistry and comparative genomic hybridisation was performed in 2003 on 25 cases initially diagnosed as retroperitoneal MFH [26]. It was found that extensive tissue sampling is necessary in cases of retroperitoneal poorly differentiated sarcomas similar to MFH or fibrosarcoma. In particular, this is to find a well differentiated liposarcomatous component within a de-differentiated liposarcoma. De-differentiated liposarcomas are radiologically and pathologically heterogenous. Uninformative sampling difficulties are a self-evident hazard. These sarcomas have juxtaposition of well-differentiated liposarcoma components with a non-lipogenic sarcoma. Immunohistochemistry for CDK4 and MDM2 that reside in the Chr. 12q13-15 region is also useful [27].

The nuclear hormone receptor, peroxisome proliferator-activated receptor gamma (PPAR-γ) is involved in adipocyte differentiation. Investigators performed immunohistochemical staining for PPAR-γ on 40 dedifferentiated liposarcomas and compared them with 24 retroperitoneal sarcomas that did not have lipogenic differentiation [28]. Specific immunostaining was present in 93 of the dedifferentiated liposarcomas and 25% (6/24) of the other sarcoma types. Of this last group, four were undifferentiated sarcomas and immunostaining for MDM2 and/or CDK4 was positive in three. It is to be inferred that these cases may have been initially, unrecognized dedifferentiated liposarcomas. Finally, it is to be recalled that MFH of bone is traditionally treated like a high grade osteosarcoma. Some clinical discriminators between MFH of bone and an osteosarcoma are that MFH of bone occurs more frequently in older patients, is more frequently osteolytic and usual is accompanied by normal serum levels of alkaline phosphatase.

## 7. New Advances in the Diagnosis of Carcinomas: A New Era That May Influence UPS Diagnostic Research

New advances offer the promise of a more effective way to find the lineage of cellular differentiation of undifferentiated pleomorphic sarcomas. In a more general advance Haun *et al.* in Boston, MA devised a point of care rapid quantitative system to diagnose subtypes of cancer [29]. Within a cohort of 50 patients with suspected malignant abdominal lesions a micro-NMR system correctly identified 44 patients as having malignant lesions using detection of their nine marker signature [EpCAM, Epidermal growth factor receptor (EGFR), vimentin, B7-H3, MUC-1, p53, (human epidermal growth factor receptor 2) HER2, CK18, and Ki-67] with the diagnosis being verified by standard techniques. Six patients had benign lesions and the remaining 44 cases were different types of carcinoma (epithelial malignancies). Obviously sarcomas and in particular undifferentiated pleomorphic sarcomas were not the subject of this study. It is however an exemplary example of how the evaluation of lesions that would previously have been classified as MFH, potentially could be reconfigured in a clinically practicable way. MUC-1 had the highest area under the receiver operator characteristic curve (plot of sensitivity *x-axis*, 1-specificity *y-axis*) A_z_ = 0.82. With particular reference to sarcomas vimentin had an A_z_ of 0.64 (A_z_ 0.5 uninformative; 0.7 good; 0.8 excellent, sensitivity and specificity). The study investigators used nanoparticle-based magnetic affinity ligands to detect the expression of malignancy associated protein markers by analysing cells obtained by fine aspiration in the 50 patients. This allowed rapid multiplexed analysis within one hour. Using the four protein (MUC-1, HER2, epidermal growth factor receptor, and EpCAM) signature the diagnostic accuracy of the system was 96%, which exceeded an accuracy of 84% for immunohistochemistry. This micro-NMR system, though more accurate and quicker than conventional methods, in liable to error because of heterogenous expression of protein marker within tumors. Proteins are also quite liable to degradation requiring immediate analysis or preservation. The incorporation of additional markers such as Cluster of Differentiation163 (CD163), CD33, CD14, CD16 and 5B5) that more accurately define monocyte macrophage and fibroblast populations would permit the characterisation of the inflammatory and stromal components. This especially may have implications for UPS.

## 8. The Importance of Finding True Lineage Differentiation in Undifferentiated Pleomorphic Sarcoma

The careful approach to cases of MFH applied by Fletcher (tumour morphology, immune-histochemistry and ultra-structural analysis) has led to attrition in the percentage of cases diagnosed as MFH, a diagnosis of exclusion. Secondly, gene clustering paradigms including: (i) Euclidean distance between data points (ii) The Pearson correlation coefficient, and (iii) Mahalanobis distance can segregate tumors based on gene expression profiles. However, interestingly in the Nakayama study a third of MFH had a similar gene expression profile with myxofibrosarcoma. Thirdly, despite the decrement in cases of MFH due to improved pathologic assessment a residual number of cases persist. This may be because the individual tumors are so pleomorphic that despite careful assessment a specific lineage of differentiation cannot be defined. This partially may be due to the lack or routine use of gene expression arrays and gene clustering algorithms in clinical practice. It also may be due to persisting insufficient knowledge of sarcoma subtypes that may be discovered in the future. For example, in 2011 a rare vascular sarcoma called epithelioid hemangioendothelioma was found to have a WWTR1/CAMTA1 gene fusion and a specific diagnostic fluorescent in situ hybridisation assay for this translocation was devised [30]. This was discovered by combined cytogenetic methods with deep transcriptome sequencing. Subsequently this was then used to find the product of the characteristic tumor chromosomal translocation (1;13)(p36;q25). These tumors were previously liable to be confused with epithelioid haemangioma, and epithelioid angiosarcomas. Unfortunately, despite improved detection of this sarcoma subtype, no treatment aside from surgery exists for this tumor.

Improved sub-categorisation of undifferentiated pleomorphic sarcoma offers further potential therapeutic opportunities. The percentage of cases of sarcoma diagnosed as undifferentiated pleomorphic sarcoma within an institutions pathology service over time is also an internal audit of the quality and extent of tissue sampling. It is also a vicarious measure of the appropriateness and extent of the applied immunohistochemistry panel. Lower rates of MFH would be expected in good performing departments. Further diminishment in the rate of MFH diagnosis would be anticipated with gene profiling and recently array-comparative genomic hybridization (a-CGH) detecting microdeletions and duplications of DNA sequences. These techniques are of academic importance rather than in routine clinical practice presently.

## 9. Modelling Sarcoma Using Mesenchymal Stem Cells

The emergent opinion is that MFH is a heterogenous disorder mainly due to contamination of this tumor category with sarcomas of other lineages. The historic perspective that it may be a malignancy of a mesenchymal cell type with a pluripotential capacity to differentiate into different lineages still deserves further critical appraisal. Minimum criteria have been proposed for defining *ex-vivo* mesenchymal stem cells in culture: (i) The cells must express CD73, CD 105 and CD90 and lack expression of CD34, CD45, CD11b, CD14, CD19, CD79b and HLA-DR; (ii) the cells when maintained in standard culture conditions must be plastic adherent; (iii) they should have the capacity to differentiate into osteoblasts, adipocytes and chondroblasts [31].

As previously detailed, within the two main subdivision of sarcoma the second group has complex karyotypes and includes MFH, leiomyosarcoma and osteosarcoma [32]. The transformation of mesenchymal stem cells can occur by loss of cell cycle regulators, but they can also be transformed by alterations in WNT and Phosphatidyl inositol 3-kinase-Protein Kinase B (PI3K-AKT) signalling. Wnt modulates the balance between self-renewal and differentiation in stem and progenitor cells [33]. The role of WNT in sarcomas differs from that of carcinomas. WNT signalling inhibition is associated with human mesenchymal stem cell transformation in sarcomagenesis. However, in models of carcinomas activating mutations in WNT pathway components are observed such as loss of APC function or oncogenic mutations in β-catenin in the majority of cases of sporadic colorectal cancer [34,35].

Research from Columbia University and Memorial Sloane Kettering Cancer Center has shown that human mesenchymal stem cells are the progenitors of undifferentiated pleomorphic sarcoma [35]. It was found that Dickkopf-related protein 1 (DDK1) a WNT inhibitor, inhibits the human mesenchymal stem cells from becoming committed to differentiation via Wnt2/β-catenin canonical signalling. Additionally Wnt5A/JNK non-canonical signalling regulates a viability check point independent of Dkk1. It was also shown that inhibition of Wnt signalling can transform human mesenchymal stem cells to form UPS like tumors in nude mice and that when Wnt signalling is appropriately re-established in UPS cells they can differentiate along mature connective tissue lineages. The potential of a therapeutic differentiation strategy for treating sarcoma analagous to 13-cis retinoic acid for high risk neuroblastoma can be inferred preclinically from these findings [36].

*In vivo* models also exist that are informative about molecular aberrations in tumors that accidently are designated as UPS. For instance, in a murine model, loss of expression of p53 in p21^−/−^ p53^+/−^ mesenchymal stem cells results in the formation of fibrosarcoma like tumors [37]. Karyotypic instability occurred accompanied by loss of p16^INK4A^ which prevents senescence. 

## 10. Analysis of Multiple Sarcoma Expression Databases

In 2010 investigators at Harvard University analysed five independent microarray databases (325 tumor arrays) with the intention of improving classification of previously unclassifiable sarcomas [8,15,17,38,39,40]. Genome wide hierarchical clustering analysis and subclass mapping was used to find a molecular “match” for MFH and NOS tumors, compared to tumors with established lineage differentiation. A 170-gene predictor was developed and independently validated. The study consisted of an iterative process using different tumor cohorts to develop the classifier, validate the classifier, re-classify MFH + NOS tumors, and diagnose unclassified sarcomas.

In an initial step, a 138 gene probes set for fibrosarcoma, liposarcoma, leiomyosarcoma, rhabdomyosarcoma and MPNST/SYN was applied. This was then followed by a second step of 35 probes for MPNST/SYN as described in Table 3 and Table 4. The model was refined using a 85 tumor case series from the National Cancer Institute (NCI). The nearest centroid prediction algorithm from the NCI dataset was externally validates on four other databases with accuracies of 78–86%. These datasets had differing combinations of fibrosarcoma, leiomyosarcoma, liposarcoma, synovial sarcoma and MPNST. When the gene predictor was applied to MFH + NOS cases, the tumors were re-classified as fibrosarcomas, leiomyosarcomas and liposarcomas. Specific marker expression could not be assigned for MFH-MPNST and MFH-Synovial sarcoma due to small numbers predicted within these categories.

Interestingly it was observes that some of the classifier genes were potential targets for targeted therapeutic development including IGF-1, PPARγ, NGF beta and FGF receptor 3 [41,42,43,44].

**Table 3:** Distinct expression patterns of the first step of the Harvard Study, a 138-gene predictor.

Distinct differentiation states	Selected differentially expressed genes
Smooth muscle differentiation genes	Myosin light chain kinase
	Calponin 1
	Smooth muscle actin γ2
	Alpha 2 macroglobulin
	Cysteine and glycine-rich protein 1
	Chromosome 9 ORF 3
Peripheral nerve differentiation genes	Jagged 1
	Glycoprotein M6B
	Olfactomedin 1
	Nerve growth factor beta
	Peripheral myelin protein 22
Fibroblast differentiation genes	Kallikrein-related peptide 7
	Lumican
	Cadherin 1
	Complement factor H
	Ficolin
	Fibroblast growth factor receptor 3
Adipocyte differentiation genes	Fatty acid binding protein 4
	PPAR gamma
	IGF-1
	Palmdelphin
	Acyl-CoA synthetase long-chain members 1 and 5

**Table 4:** Distinct expression pattern in the second step of the Hardvard Study, a 35 gene classifier.

Distinct differentiation states	Selected differentially expressed genes
Synovial sarcoma genes	Synovial sarcoma, X Breakpoint 1
	Cyclin D1
	BCL2
	Vitronectin
	Cell adhesion molecule 1
	RIMS binding protein 2
Malignant nerve sheath genes	Synuclein alpha
	Synaptotagmin 17
	Transducin beta like 2
	Intercellular adhesion molecule 3
	S100 calcium binding protein A3
	Dual specific phosphatase 4

## 11. Undifferentiated Pleomorphic Sarcoma the Past and the Future

In the 2007 gene expression study of Nakayama the sarcoma subtype of MFH was found to be contaminated with other lineages such as myxofibrosarcoma, leiomyosarcoma, dedifferentiated liposarcoma, fibrosarcoma and malignant peripheral nerve sheath tumor. Myxofibrosarcoma is now considered separately in the AJCC classification system. Leiomyosarcomas have an inferior prognosis to “true” cases of MFH, and as shown by Baird in 2005, MFH in addition to being next to leiomyosarcoma in the gene cluster dendrogram has within it a subset dominated by genes of myogenic differentiation. Therefore, in addition to providing therapeutic guidance this information has prognostic importance. In Fletchers’ 1992 retrospective review he used diagnostic criteria for leiomyosarcoma that included, co-expression of desmin and actin with myoglobin negativity and an absence of cross striations. While more extensive and meticulous tissue sampling is of prevailing importance with respect to all potential contaminating subtypes it is particularly the case for detection of de-differentiated liposarcoma. Detection of amplification of MDM2 and/or CDK4 is potentially informative with respect to this sarcoma type.

A cross species genomic analysis has led to the identification of a mouse model of undifferentiated pleomorphic sarcoma [45]. A gene-set from the LSL-Kras^G12D^; p53^Flox/Flox^ murine model of soft tissue sarcoma was found to be highly enriched in undifferentiated pleomorphic sarcoma. The RAS target FOXM1 was identified using this gene-set in human undifferentiated pleomorphic sarcoma. Within the mouse model *Foxm1* was elevated in those sarcomas that metastasised to the lung. This study is a fascinating advance demonstrating the utility of this mouse model for further investigation of the pathogenesis of human undifferentiated pleomorphic sarcoma. Furthermore, direct targeting of RAS is notoriously difficult. Fifteen percent of cutaneous melanoma have mutations of RAS. NRAS mutant melanoma is mutually exclusive of BRAF mutant melanoma. In 2012 an inducible mouse model of NRAS mutant melanoma and network modelling established the *in-vivo* therapeutic syngeristic efficacy of targeted inhibition of CDK4 and MEK to decrease melanoma survival and proliferation [46]. Perhaps, the methodologies used in this preclinical study of NRAS mutant melanoma may be used to evalute a strategy of vicarious RAS targeting to treat sarcomas with elevated *Foxm1*. 

In a chemically inducible rat sarcoma model a comparative analysis of gene expression profiles in osteosarcomas and MFH was undertaken to identify tumourigenic signaling pathways [47]. Mismatch repair, DNA repair and cell cycle pathways as well as Hedgehog signaling was upregulated in both types of sarcoma. The Smothened receptor anatagonist Vismodegib which inhibits Hedgehog signaling is already approved for treating locally advanced and metastatic basal cell carcinomas [48,49]. Within the inducible rat sarcoma model of osteosarcoma and MFH, pathways involved in cell adhesion, cytokine receptors, extracellular matrix receptors and Wnt signalling were down regulated. Re-establishment of Wnt signalling in UPS is a therapeutic aspiration that will probably require virus vector strategies if it is ever to be fulfilled.

The afore-mentioned UPS mouse model was also used is a small study that evaluated doxorubicin and inhibitors of the PI3K pathway [BKM120 (PI3K inhibitor) and BEZ235 (a dual PI3K/mTOR inhibitor)]. Doxorubicin treatment conferred a partial response in 6.6% of treated tumors, whereas the combination of BEZ235 and doxorubicin conferred a complete response rate of 50% (*p* = 0.035). The inference of these findings is that PI3K pathway inhibition is potentially efficacious in treating soft tissue sarcoma. As the gene set from the LSL-Kras^G12D^; p53^Flox/Flox^ murine model was highly enriched for undifferentiated pleomorphic sarcoma, it may arise that this inference is particular pertinent for undifferentiated pleomorphic sarcomas. Finally, an M.D. Anderson study found that p-MEK and p-AKT were correlated on multi-variate analysis with an adverse prognosis [50]. These findings in conjunction with further immunohistochemistry inferred potential therapeutic promise from targeting HGF, c-Met, MEK/extracellular-regulated kinase (ERK) and/or AKT in a subgroup of UPS/MFH patients.

## 12. Conclusions

The benefit that can be derived from accurate characterization of the biology of sarcomas is exemplified by treating gastrointestinal stromal tumors with molecules that inhibit c-KIT and PDGFRA. Clarification of the heterogenous diagnosis of “UPS” into specific sarcoma subtypes is important. It allows patients to receive specific treatments such as trabecidin in myxoid/round cell liposarcoma. It also may by a process of attrition in the future, reveal new residual sarcoma subgroups that have eluded recognition so far. A once mysterious enemy may just be comprised of familiar foes or a new adversary may emerge from amongst them.
